# CVT-Based Asynchronous BCI for Brain-Controlled Robot Navigation

**DOI:** 10.34133/cbsystems.0024

**Published:** 2023-04-18

**Authors:** Mengfan Li, Ran Wei, Ziqi Zhang, Pengfei Zhang, Guizhi Xu, Wenzhe Liao

**Affiliations:** ^1^ State Key Laboratory of Reliability and Intelligence of Electrical Equipment, School of Health Sciences and Biomedical Engineering, Hebei University of Technology, 300132 Tianjin, China.; ^2^ Hebei Key Laboratory of Bioelectromagnetics and Neuroengineering, 300132 Tianjin, China.; ^3^ Tianjin Key Laboratory of Bioelectromagnetic Technology and Intelligent Health, 300132 Tianjin, China.; ^4^ School of Artificial Intelligence, Hebei University of Technology, 300132 Tianjin, China.

## Abstract

Brain–computer interface (BCI) is a typical direction of integration of human intelligence and robot intelligence. Shared control is an essential form of combining human and robot agents in a common task, but still faces a lack of freedom for the human agent. This paper proposes a Centroidal Voronoi Tessellation (CVT)-based road segmentation approach for brain-controlled robot navigation by means of asynchronous BCI. An electromyogram-based asynchronous mechanism is introduced into the BCI system for self-paced control. A novel CVT-based road segmentation method is provided to generate optional navigation goals in the road area for arbitrary goal selection. An event-related potential of the BCI is designed for target selection to communicate with the robot. The robot has an autonomous navigation function to reach the human selected goals. A comparison experiment in the single-step control pattern is executed to verify the effectiveness of the CVT-based asynchronous (CVT-A) BCI system. Eight subjects participated in the experiment, and they were instructed to control the robot to navigate toward a destination with obstacle avoidance tasks. The results show that the CVT-A BCI system can shorten the task duration, decrease the command times, and optimize navigation path, compared with the single-step pattern. Moreover, this shared control mechanism of the CVT-A BCI system contributes to the promotion of human and robot agent integration control in unstructured environments.

## Introduction

The brain–computer interface (BCI) is a novel communication and control technology that establishes an information output pathway between the human brain and other electronic devices, not relying on conventional brain information output pathways (peripheral nerves and related muscle tissues) [1,2]. The typical electroencephalography (EEG) models with respect to brain signal generation in BCIs include motor imagery (MI) [3], event-related potentials (ERPs) [4], and steady-state visual evoked potentials (SSVEPs) [5]. As a new type of human–machine interaction (HMI) technology, BCI has received increasing attention. It has been employed in the military, the medical field, the technological field, and households [6–9]. Unlike traditional HMI technologies that manipulate electronic devices through keyboard, mouse, joysticks, voice, and gestures [10], BCI actively understands human intentions [11]. BCI has great application potential for the disabled community [12,13] and groups that require extended interaction capabilities, such as brain-actuated functional electrical stimulation to achieve motor recovery after stroke [14]. The brain-controlled robotic arm assists patients with motor disabilities to grasp objects [15].

It is well known that robot agents are adept at handling complex tasks with powerful analysis and calculation capabilities, while human agents excel at designing and reasoning to deal with uncertain, marginal problems. This generates the thoughts of shared control in the BCI domains, which incorporates human control commands decoded by EEG and robot control commands obtained by machine intelligence, fusing both commands at different levels. One of the important applications of shared control is to improve impaired physical functions of people with disabilities, such as motor functions [16,17] or speech functions [18,19]. Shared control combines with machine vision [20], indoor location [21], automatic navigation [22], and other assistive technologies that greatly improve the intelligence and accuracy of the BCI-based control system. Xu et al. [20] utilized robot vision to identify and locate the potential targets and grasp automatically. Duan et al. [23] designed a hybrid BCI system that can provide multimodal BCI control commands to the visual servo module. Liu et al. [24] used the robot's equipped Kinect camera and radar to implement simultaneous localization and mapping (SLAM) and the captured video stream as feedback to monitor the robot's operation. In general, the shared-control-based BCI system evolves toward making full use of the sensor resources, adding intelligent image processing algorithms, and applying hybrid signal-based BCI.

Generally, the shared-control-based BCI system is categorized as 2 strategies from the perspective of human agent and robot agent participation time, namely, time-shared control strategy [25,26] and all-time shared control strategy [27,28]. In the all-time shared control strategy, the human agent and robot agent simultaneously collaborate to control the robot during the control cycle. Yuan and Li [29] proposed a polynomial trajectory strategy based on EEG signals to ensure the continuity of wheelchair trajectory and combine angle and visual localization to control robot walking. Ezeh et al. [28] designed a probabilistic shared control algorithm that combines human intention probability-based input velocity and robot probability-based planning velocity to reduce collisions. Frequent command output increases the burden on the human brain in the all-time shared control system, and enhancing system intelligence also poses a challenge to the system design.

In the time-shared control strategy, the human agent and robot agent work at different periods. The human agent determines the target object or destination and the robot agent is responsible for performing the task automatically or is triggered only under specific conditions [30]. Due to fewer EEG command outputs, the time-shared control strategy can reduce the burden of human operation and improve the efficiency of user operation. Mao et al. [31] extracted objects as path nodes, controlling the robot navigation autonomously to the nodes selected by the user. Tang and Zhou [32] identified and located the target water bottle and fed it into the ERP paradigm for the user to choose. However, the user is not able to set targets in free space; instead, the robot would only stop when it docks at a potential shapely target. The selection of target is dependent and incomprehensive.

For the purpose of arbitrary target selection, an object-independent target generation method is needed. Centroidal Voronoi Tessellation (CVT) is a method of partitioning 2D or 3D space and is mainly used in the areas of resource allocation [33], facility location [34], image segmentation [35], etc. Soni and Bhowmick [36] used the CVT approach to generate quadrangular mesh on smooth surface modeling. It can partition the plane into several polygonal regions like cell arrangement, which is suitable for solving the partitioning problem of irregular environmental area. In this study, the CVT method is applied to segment road areas into several cells. The user selects goals from a set of generated cells in the environment that are safely and autonomously reached by the autonomous robot system. Therefore, this system achieves object-independent target selection by the CVT approach.

The human agent and robot agent are controlled in separate phases in the time-shared control system, hence limiting the human's freedom to intervention and thus needs to provide more interaction to improve the shared control strategy. Therefore, a speedy and robust intervention mechanism is needed. In an asynchronous BCI system, humans, rather than the computer, determine when to issue the BCI commands [15]. Thus, subjects can freely intervene in the control system as they wish. Zhou et al. [37] utilized high-frequency SSVEP-BCI as a state switching interface. Yu et al. [38] utilized the MI signal as a basis for state detection to switch from the control state to the idle state. The judgment basis for distinguishing brain states is a key point to achieve an efficient and rapid state switch. Physiological signals such as electromyogram (EMG) or electrooculogram (EOG), or a new EEG evoking paradigm is usually utilized for state detection. In practice, human intervention, which is the detection of the control state, must be elicited as promptly as possible, in order to avoid missing the nodes that humans want to control. Lin et al. [39] compared pure hybrid BCI, EOG-based hybrid BCI, and EMG-based hybrid BCI, where EMG-based hybrid BCI yielded the highest information transfer rate (ITR). In this study, the EMG acquired by clenching teeth is employed to distinguish between the control state and the idle state for asynchronous control.

In this study, a CVT-based asynchronous (CVT-A) BCI system is proposed to efficiently control the robot navigation in a self-paced manner. A novel CVT-based road segmentation method is used to achieve object-independent target selection in the environment. The asynchronous control mechanism combines EMG and EEG, which allows subjects to switch between the idle state and the control state and select targets for autonomous robot navigation. The targets in the ERP paradigm are derived from the environmental information perceived by the depth camera on the robot. Due to the free human intervention, the system is adaptive to the environment with special obstacles (e.g., extremely low obstacles). Eight healthy subjects accomplished the navigation experiment with obstacle avoidance. The aim of this study is to provide a free asynchronous control mechanism and an arbitrary target selection method, which proves that hybrid signal fusion and computer vision improve the real-time nature and intelligence for the BCI field.

## Materials and Methods

### Architecture overview

**Fig. 1.**
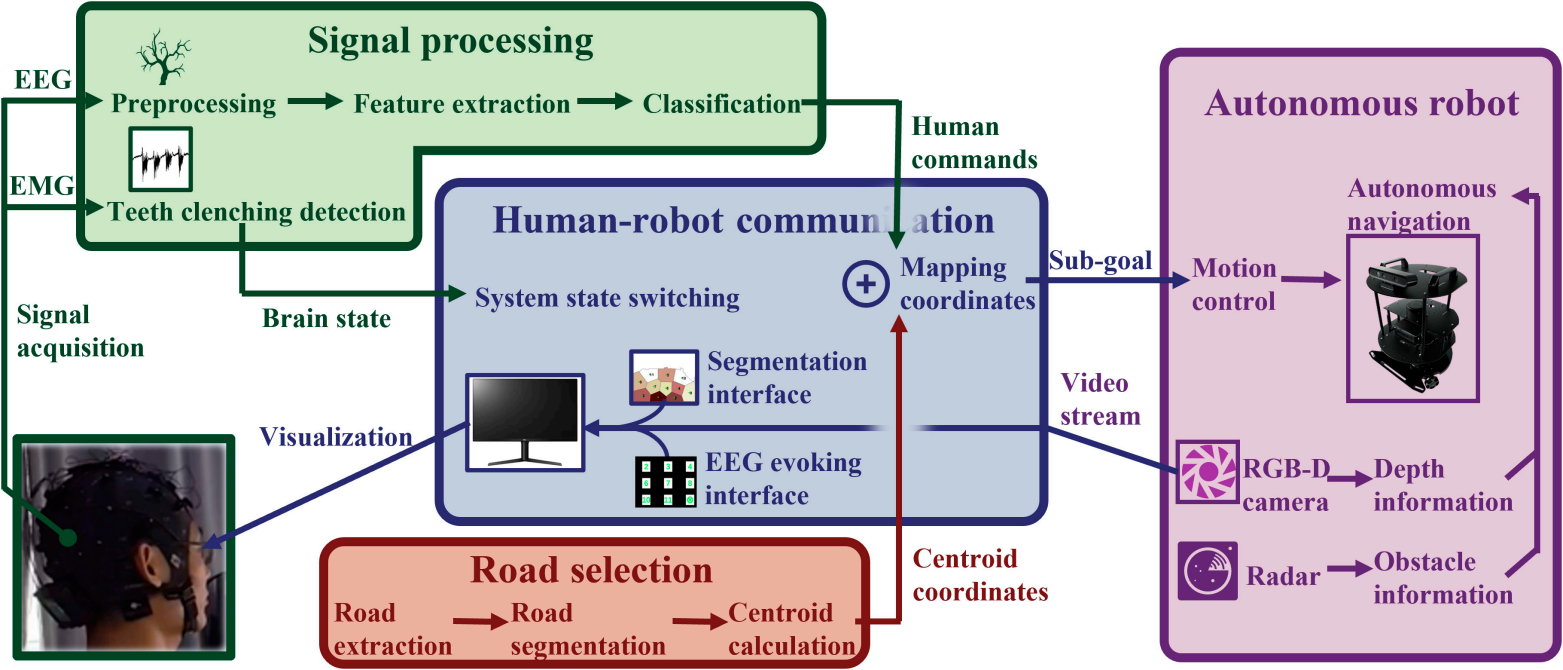
The architecture of the CVT-A BCI system.

The CVT-A BCI system consists of 4 main subsystems as shown in Fig. 1: a signal processing system, a human–robot communication system, a road selection system, and an autonomous robot system. The signal processing system is in charge of acquiring EMG and EEG for decoding the signals to distinguish the control state or the idle state and control robot motion. The human–robot communication system is responsible for giving commands to the other 3 subsystems, displaying user interface (UI) for subjects to monitor system states and select commands. The road selection system acquires environmental images by robot and converts them into sub-goal coordinates for projecting to the human–robot communication system. The autonomous robot receives commands from the human–robot communication system and performs trajectory planning and motion control.

### EMG-based brain state monitoring and EEG-based command output

#### EMG signal monitoring

**Fig. 2.**
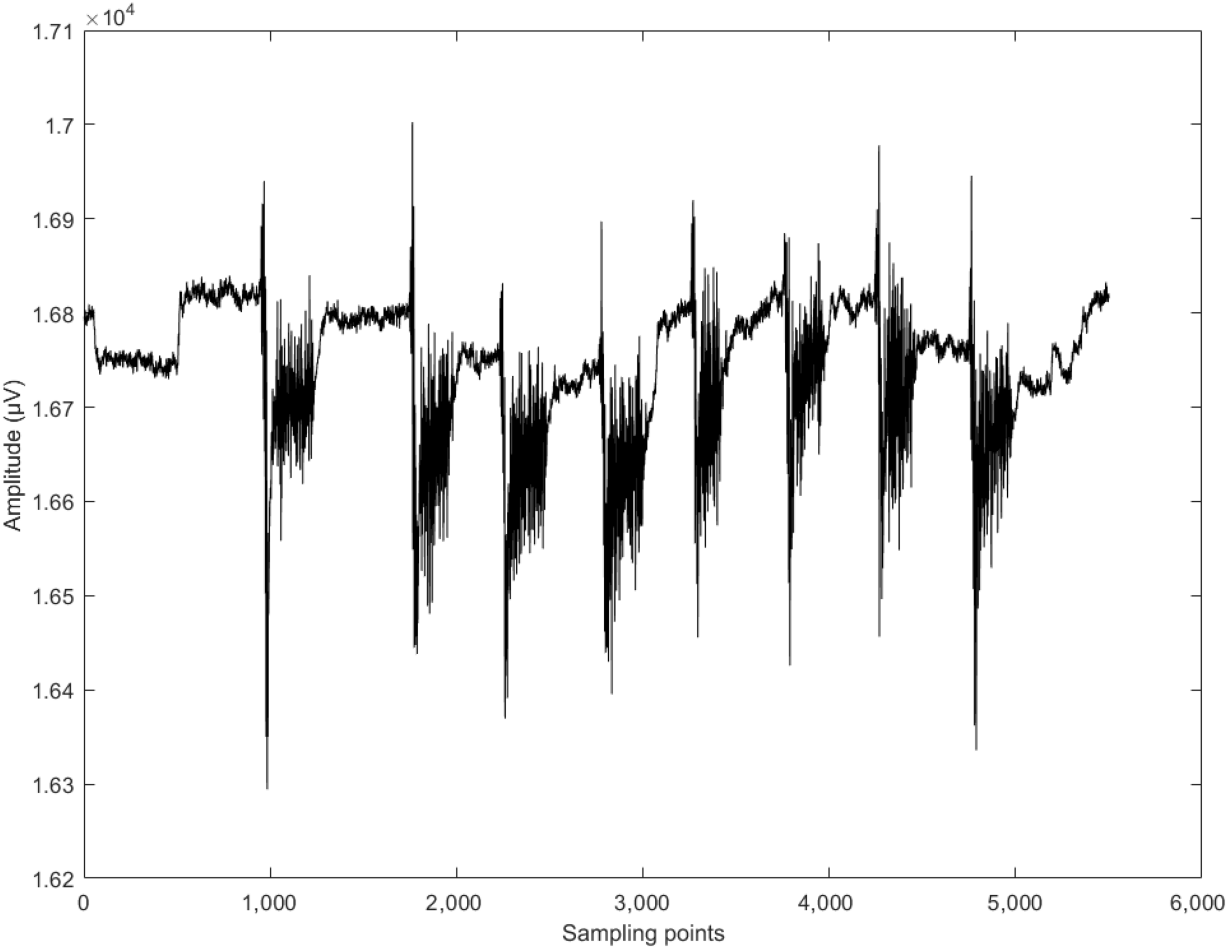
The EMG signals of channel FC5 performing teeth clenching 8 times.

The EMG signals are acquired and processed in real time during the idle state, and act as a “brain state switch” to switch over from the idle state to the control state. The source of EMG is produced by clenching teeth and is recorded by an EEG and EMG acquisition device (g.Nautilus, 32 channels) in the FC5 channel with a sampling rate of 250 Hz. Calculating the brain state requires computing the peak-to-peak value of EMG amplitude in a sliding time window of 1 s without overlap in a signal analysis computer (Intel Core i7-8750H, 2.2 GHz). The control state is triggered when the peak-to-peak value is greater than a subject-specific threshold. Once the control state is triggered, EMG acquisition and processing stops and every subsystem begins to switch state. Figure 2 demonstrates the EMG signal amplitudes for 8 clenching behaviors; the horizontal axis denotes sampling points and the vertical axis indicates amplitude.

The subject is asked to execute several teeth clenching for threshold calibration before the online experiment. The threshold is set to ensure sensitive triggering without being affected by other EMG activities such as normal speech and swallowing. The EMG data analysis script displays the amplitude plot in real time on the screen, allowing better optimization of the threshold based on the screen value. Teeth clenching trigger flag *F*(*e_i_*) is calculated as follows:

$$F\left(e_{i}\right)=\left\{\begin{array}{c} 1,\max\left\{e_{i}\right\}-\min\left\{e_{i}\right\}\geq T_{0}\\ 0,\max\left\{e_{i}\right\}-\min\left\{e_{i}\right\}<T_{0} \end{array}\right\}.$$ (1)

where *e_i_* refers to the value of the of EMG signals at sample point *i*, max{*e_i_*} refers to the maximum value in a sampling time window, and min{*e_i_*} refers to the minimum value in a sampling time window. *T*_0_ refers to the value of the subject-specific threshold, and the time-window size P is 250. When the value of *F*(*e_i_*) is equal to 1, teeth clenching is regarded as detected and triggers the control state; when the value of *F*(*e_i_*) is equal to 0, it remains in the idle state.

#### BCI graphical UI and commands

The BCI system uses a visual oddball paradigm to evoke ERP to control the robot. The visual interface (1,920 × 1,080 pixels, 50 Hz refresh rate) displays a 3 × 4 image matrix to the subject and the images flash randomly in the form of rows and columns. Each image in the ERP evoking interface contains 2 elements, which is one arrow marker and one numeric marker, and the details are shown in Table 1. The arrow markers pointing to different directions are suitable as single-step motion commands, as the graphical element allows subjects to capture the image information quickly. The numeric markers in different numbers are suitable for assigning each number a meaning, as it can represent multiple meanings at different environmental contexts. In this study, the arrow markers indicate commands to control the single-step movements of the robot, and the numeric markers represent sub-goal commands controlling the robot to reach automatically.

**Table 1.** ERP evoking interfaces containing 2 elements.

Image Position	Column 1	Column 2	Column 3	Column 4
Row 1	Turn left 30°	Move to the left front 20 cm	Move to the right front 20 cm	Turn right 30°
	Number 1	Number 2	Number 3	Number 4
Row 2	Move forward 40 cm	Move to the left 20 cm	Move to the right 20 cm	Move backward 20 cm
	Number 5	Number 6	Number 7	Number 8
Row 3	Move forward 20 cm	Move to the left rear 20 cm	Move to the right rear 20 cm	Exit
	Number 9	Number 10	Number 11	Exit

The stimulus onset asynchrony is set to 140 ms, which includes highlighting one row or column for 100 ms and shielding this row or column for 40 ms. The process in which all rows and columns are presented once is a repetition; thus, each target flashes twice in a single repetition. The target is the stimulus image corresponding to the subject's command, and the nontarget is the stimulus that the subject should ignore. Several repetitions constitute a trial in which the subject needs to focus on one target stimulus from beginning to end. In our experiment, 10 repetitions compose an offline trial and 5 repetitions compose an online trial.

**Fig. 3.**
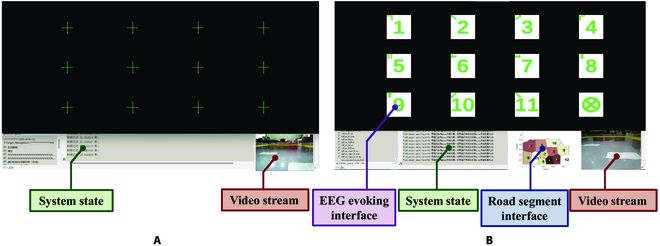
The UI in different brain states. (A) UI in the idle state. (B) UI in the control state.

The UI presents different contents for the subject to monitor or focus in different brain states. It consists of a video stream and a system operation state for the subject supervising in the idle state. The ERP evoking interface is shielded by a green plus sign as shown in Fig. 3A. During the control state, the UI presents an ERP evoking interface for subjects to focus on target command based on the system operation state. The system state and video stream are also presented as feedback, and the road segmentation interface offers as the basis for target command selection. The details are shown in Fig. 3B.

#### EEG signal decoding

In the process of processing the ERP signal, the acquired EEG signals are used to train the classifiers of different subjects. The EEG signals are first passed through a common average reference space filter to obtain data from 50 to 750 ms after stimulation, and then filtered by a third-order Butterworth filter (0.01 to 30 Hz). The data are downsampled to 35 Hz to obtain a 25-dimensional feature vector from each channel. Four channels (PO4, PO7, PO8, and Oz) are used as feature channels to generate a final 100-dimensional feature vector as the input to the classifier.

The FLDA is used to calculate the 100-dimensional feature vector, which groups the vectors with the same features into a class. It classifies a feature vector by the following equation:

$$f\left(x\right)=w^{T}x+w_{0}$$ (2)

where *w^T^* is the feature matrix, *x* is the input vector, and *w*_0_ is a threshold. The feature vector belongs to the positive class if *f*(*x*) is positive; otherwise, it belongs to the negative class. If it is a positive class, it is deemed a target command; otherwise, it is deemed a non-target command.

### Autonomous robot

**Fig. 4.**
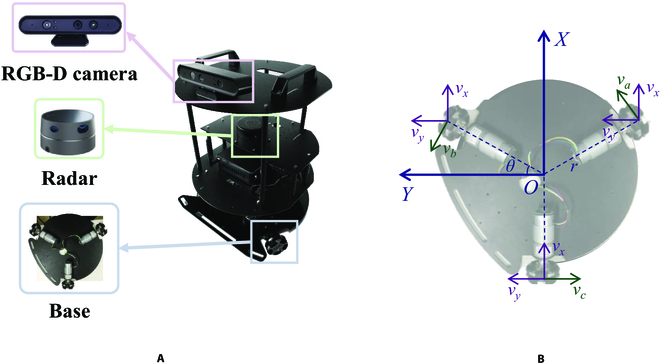
(A) Configuration of the robot. (B) Robot motion model.

The robot (MCU: MC9S08AC16CFGE, i3-7100U) is an automatic system that receives and executes control commands from the human–robot communication system in the control state, while it reaches the destination autonomously in the idle state. The structure of the robot motion model with chassis radius *R* = 0.2 m and length *L* = 0.7 m is shown in Fig. 4A. A radar is equipped at a height of 0.3 m to build maps and perceive obstacles, with a 360° scanning range and a 0.1 to 0.8 m detection range. An RGB-D camera containing an RGB camera and a depth camera is mounted at a height of 0.65 m to capture video stream and depth information from the environment, with a 70° horizontal viewing range, a 60° vertical viewing range, and a 1.2 to 3.5 m depth range. The robot has 3 motor-driven omnidirectional wheels exhibiting a 120° angle to each other that allows the robot to travel in all directions. The kinematic matrix of the robot is as follows.

$$\begin{array}{c} \left[\begin{array}{c} v_{a}\\ v_{b}\\ v_{c} \end{array}\right]=\left[\begin{array}{c} \text{cos}\theta \\ -\text{cos}\theta \\ 0 \end{array}\;\begin{array}{c} \text{sin}\theta \\ \text{sin}\theta \\ 0 \end{array}\;\begin{array}{c} r\\ r\\ r \end{array}\right]\left[\begin{array}{c} v_{x}\\ v_{y}\\ \theta \end{array}\right]. \end{array}$$ (3)

where *v_a_*, *v_b_*, and *v_c_* represent the linear velocity of the wheel, respectively. *v_x_* and *v_y_* represent the linear velocity of the robot in the *X* and *Y* directions, respectively. *r* is the vertical distance from the center of the robot to the wheel, representing the radius of rotation. *θ* is an angle of 30°. The motion model is shown in Fig. 4B.

The autonomous robot system senses the external environment through sensors and navigates autonomously to the destination. The radar-based SLAM method is used to navigate toward the destination in the idle state and build a 2D grid map of the environment. The SLAM contains a Gmapping algorithm that is a particle filtering algorithm for building 2D grid maps based on radar data. The vision-based navigation method is utilized to reach the sub-goal issued by ERP commands in the control state. The robot operating system (ROS) is integrated in the robot, whose version is Kinetic Kame.

### CVT-based road selection

When the system switches to the control state, the road selection subsystem starts to execute CVT-based road selection. Road selection and human–robot communication subsystems operate simultaneously on the same computer (Intel Core i7-9700, 3.0 GHz). Road selection is applied to segment and locate the road area for the subject’s sub-goal selection when the robot cannot avoid obstacles by itself. In the road selection subsystem, the road area ahead the robot is extracted first and then a novel CVT approach is used to segment the road area and calculate the centroid. The detail of the road segmentation progress is as below.

#### Road extraction

**Fig. 5.**
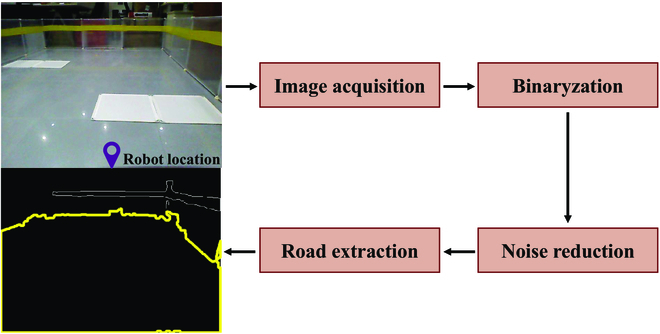
The process of road area extraction.

Road extraction is the first step in the process of road selection to obtain all optional sub-goals ahead of the robot. Environmental information is acquired by the RGB-D camera with a 30° downward view. The classical K-means algorithm is used to cluster the RGB color space of an image in 2 classes, to obtain a binarized image that is discrete and multi-regional. Then image-close and image-open is applied to eliminate the small isolated areas in the binarized image for the purpose of noise reduction. Because the robot's camera is angled downward, the area under the robot's feet that is located in the middle of the bottom border in the image should theoretically be the road area. The boundaries of all discrete areas are calculated by detecting the pixel values in the binarized image after noise reduction, where the middle of the bottom border contains the road area boundary, as shown in Fig. 5.

#### CVT-based road segmentation

Road segmentation serves as the second procedure of road selection to generate the sub-goal. A CVT method is used to segment the road area and solve for the centroid. The Voronoi tessellation consists of a set of continuous polygons formed by the perpendicular bisector of a line joining 2 neighboring points. The CVT is a special Voronoi tessellation whose generator coincides with the centroid of each Voronoi cell.

In this study, it is given arbitrary *n* points called generators on the road area. According to the position of generators, the road area will be divided into *n* Voronoi cells and all cells are disjoint and cover the whole road domain. The distances from all points in the Voronoi cell to their generator are minimized compared to the distances to other generators.

Given a set of generators ${\left\{x_{i}\right\}}_{i=1}^{n}$ belonging to road area Ω, the Voronoi cell *V_i_* corresponding to the generator *x_i_* is defined as:

$$V_{i}=\left\{y\in \Omega |\;\|y-x_{i}\|<\|y-x_{j}\|,\;j=1,\ldots ,n,\;j\neq i\right\}.$$ (4)

Given a density function *ρ* of the Voronoi cell $V\in {\left\{V_{i}\right\}}_{i=1}^{n}$, the centroid *x*^∗^ of the Voronoi cell is defined as:

$$x^{*}=\frac{\int_{V_{i}}\mathrm{y\rho}\left(y\right)\mathrm{dy}}{\int_{V_{i}}\rho \left(y\right)\mathrm{dy}}.$$ (5)

In the situation where

$$x_{i}^{*}=x_{i},i=1,\ldots ,n,$$ (6)

the generators *x_i_* for the Voronoi cells *V_i_* are themselves the centroids of those regions. We call such a tessellation a CVT. The objective function of the optimized Voronoi tessellation is defined as:

$$J\left(X,V\right)=\sum_{i=1}^{n}\int_{y\in V_{i}}{\left\|y-x_{i}\right\|}^{2}\mathrm{dy}.$$ (7)

Obviously, the objective function in the Voronoi cell is minimized when the generator and the centroids coincide. In this study, generators are randomly initialized in the road area and will gradually converge to the centroid by Lloyd's descent method, which is a classical method to constructing a CVT. The details are as follows:

a. Randomly select an initial set of *n* generator ${\left\{x_{i}\right\}}_{i=1}^{n}$.

b. Construct the Voronoi tessellation ${\left\{V_{i}\right\}}_{i=1}^{n}$ of Ω associated with the points ${\left\{x_{i}\right\}}_{i=1}^{n}$.

c. Compute the centroids of the Voronoi regions ${\left\{V_{i}\right\}}_{i=1}^{n}$ found in step a; these centroids are the new set of points ${\left\{y_{i}\right\}}_{i=1}^{n}$.

d. If this new set of points coincide with the corresponding centroids, terminate; otherwise, move all generators to their corresponding centroids and return to step b.

The road image coordinate system is different from the world coordinate system. After obtaining the pixel coordinate of the centroids, it should be converted to coordinates in the world coordinate system. The centroid coordinates should be converted to the robot coordinate system first, and then it can be converted to the world coordinate system. Finally, the *n* centroids in the CVT image correspond to the *n* positions in the road area. Therefore, it is possible to project *n* sub-goals in the road area to *n* numeric commands in BCI.

### Flow of the CVT-A BCI system

**Fig. 6.**
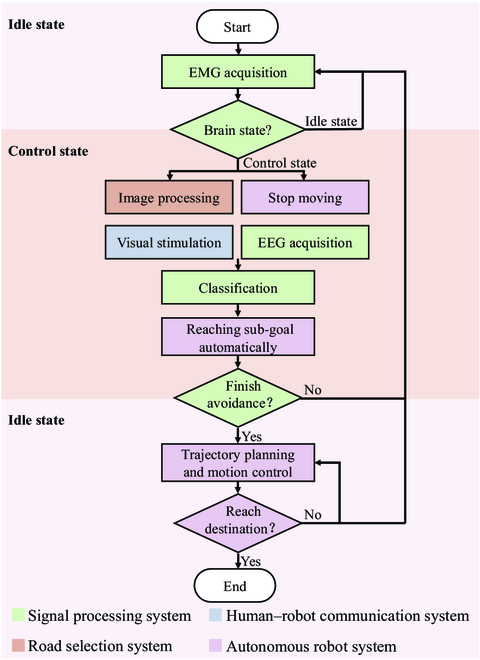
Flowchart of the CVT-A BCI system.

The CVT-A BCI system permits the subject to asynchronously assist the robot navigation by means of the user executing high-level decisions and the robot performing low-level control. The flowchart of this system is shown in Fig. 6. The EMG acquired by clenching teeth serves as a “brain state switch” to switch over from the idle state to the control state. In the idle state, the robot autonomously performs trajectory planning and motion control. In the control state, the subject sets the sub-goal for the robot to reach automatically.

At first, the subject launches a navigation task as the robot autonomously moves toward to a predefined destination. The subject monitors the robot's movements through video stream and should clench teeth on situations where the robot cannot avoid obstacles autonomously. The signal processing system cyclically detects whether the subject clenches teeth by the EMG signals. Once it is detected, the robot stops moving, the road selection system generates sub-goals, the human–robot communication system presents visual stimulation, and the signal processing system records EEG. In this way, the subject’s decisions are converted into robotic avoidance behavior through robot intelligence. Ultimately, the subject assists the robot accomplishing obstacle avoidance tasks. After that, the robot automatically navigates while monitoring the brain state until the destination is reached.

### Navigation experiment

#### Navigation scenario

**Fig. 7.**
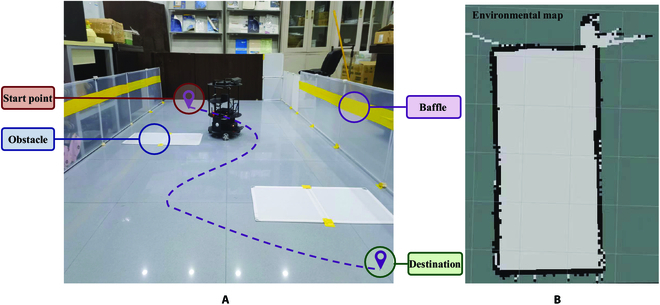
The experimental scenario. (A) Realistic experimental field. (B) Environmental map built by radar.

The field for robot navigation is 460 cm × 225 cm, enclosed by 80-cm-high baffles as shown in Fig. 7A. Two 35 cm × 60 cm extremely low obstacles are distributed in the field that cannot be scanned by radar. The mobile robot needs to move from the start point in the upper left corner of the field to the destination in the lower right corner. Brain control intervention is required to avoid obstacles that the robot cannot circumvent. The ideal movement path is shown as the purple dashed line in Fig. 7A. The environmental map built by radar is shown in Fig. 7B.

#### Online navigation experiment

The experiment invited 8 subjects (5 males and 3 females, ages from 22 to 34) who have normal or corrected-to-normal eyesight with healthy bodies, and signed written informed consents to participate in the experiments. The EEG acquisition device used the g.Nautilus wireless EEG acquisition device with a sampling rate of 250 Hz. This study adopted 6 channels (FC5, Cz, PO4, PO7, PO8, and Oz) arranged based on the 10–20 international system to record EEG and EMG. The impedance of the electrodes was kept below 5 kΩ for all electrodes. Reference electrodes were placed at the bilateral mastoids and ground electrode was placed at AFz.

To validate the performance of the proposed CVT approach applied to the asynchronous BCI system, a comparison experiment was conducted that required subjects to control the motion of the robot by a single-step pattern in an asynchronous BCI system. This study distinguishes the 2 control patterns with a single-step pattern (SS pattern) and a CVT pattern. The main distinction between them is that SS controls the robot's single movement and CVT assigns the robot's navigation goal.

SS pattern: The subject controls the single-step motion of the robot by the arrow marker commands in the ERP evoking interface. The initial state of the robot involves performing the navigation task from the start point to the destination. When an obstacle is encountered that the robot could not avoid autonomously, the subject switches to the control state by clenching teeth. Then, the subject controlled the robot via the arrow marker commands introduced in the BCI graphical UI and commands section to avoid the 2 obstacles. After avoiding obstacles, the subject can exit the control state by an exit command, and the robot navigates to the destination automatically. In this pattern, the subject selects motion commands one by one to avoid obstacles.

CVT pattern: The subject assigns a navigation goal to the robot by the numeric marker commands in the ERP evoking interface. The initial state of the robot involves performing the navigation task from the start point to the destination. When an obstacle is encountered that the robot could not avoid autonomously, the subject switches to the control state by clenching teeth. Then, the subject selects a sub-goal for the robot via the numeric marker commands introduced in the BCI graphical UI and commands section. Each numeric marker corresponds to a location of the road area in the real environment, which is generated by the CVT approach. The subject is asked to select the appropriate command, after which the robot automatically navigated to the corresponding location. After avoiding obstacles, the subject can exit the control state by an exit command, and the robot replans the path to complete the navigation to the destination. In this pattern, the subject selects target commands for the robot to execute; thus, it can achieve high control efficiency.

For the online experiment, each subject was asked to complete the same navigation task 3 times in 2 patterns successively. The navigation performance of 2 patterns was compared according to task duration, command times, path length, collision times, effort level, and trajectory. Additionally, the mean value and standard deviation (SD) among all subjects were also calculated.

The ITR is used to evaluate the output speed of commands. The equation of the ITR is as follows:

$$\mathrm{ITR}=\left\{{\text{log}}_{2}Q+p\times {\text{log}}_{2}p+\left(1-p\right)\times {\text{log}}_{2}\frac{\left(1-p\right)}{Q-1}\right\}\times \frac{60}{T}⊲$$ (8)

T=D×n+t. (9)

where *Q* is the number of visual stimuli in the interface and is set to 12; *p* is the average accuracy of each subject; *T* (seconds/target) is the time for a selection. *n* is the number of repetitions per trial; *D* is the duration of a repetition and is 0.98 s; *t* is the interval to switch from one trial to the next and is set to 0.5 s.

The task duration, command times, path length, and collision times are defined as the average number of every subject’s experiment 3 times in each pattern. The effort level is a subjective score from 1 to 5 given by subjects after completing the entire experiment, for evaluating the labors of SS and CVT patterns. Six navigation experimental trajectories (3 in SS pattern and 3 in CVT pattern) of each subject are recorded.

## Results

### Accuracy and ITR of the offline experiment

All subjects were first instructed to perform the offline data collection to train the FLDA classifier. Table 2 shows the offline accuracy (ACC) of FLDA and ITR with different repetitions. The higher the ITR, the more instructions the system can be sent out in a unit time. For one repetition, the highest ACC and ITR yield 91.7% and 43.5 bits/min by 4 subjects, while the lowest ACC and ITR result in 75.0% and 28.8 bits/min by 2 subjects.

**Table 2.** ACC and ITR of offline training in different repetitions.

Subjects	One repetition		Five repetition		Ten repetition	
	ACC (%)	ITR (bits/min)	ACC (%)	ITR (bits/min)	ACC (%	ITR (bits/min)
S1	91. 7	43.5	100.0	27.2	100.0	16.8
S2	83.3	35.5	100.0	27.2	100.0	16.8
S3	75.0	28.8	91.7	21.9	100.0	16.8
S4	75.0	28.8	100.0	27.2	100.0	16.8
S5	91.7	43.5	100.0	27.2	100.0	16.8
S6	91.7	43.5	100.0	27.2	100.0	16.8
S7	91.7	43.5	100.0	27.2	100.0	16.8
S8	83.3	35.5	100.0	27.2	100.0	16.8
Mean	85.4	37.8	99.0	26.5	100.0	16.8
SD	±7.4	±6.6	±2.9	±1.9	±0.0	±0.0

As the repetitions increase, the ACC increases for all subjects. All subjects except S3 achieve 100% in ACC and 27.2 bits/min in ITR for 5 repetitions. When the repetition times is small, variability is shown between individuals in ACC and ITR. The mean ACC and ITR of all subjects are also calculated in Table 2. The mean ACC and mean ITR are 85.4% ± 7.4% and 37.8 ± 6.6 bits/min, 99.0% ± 2.9% and 26.5 ± 1.9 bits/min, and 100.0% ± 0.0% and 16.8 ± 0.0 bits/min in 1 repetition, 5 repetitions, and 10 repetitions, respectively. Therefore, 5 repetitions were selected in the online experiments to trade off between ACC and ITR for achieving higher classification accuracy and less time. The offline experiment demonstrated the desired results, ensuring reliable and safe control of the robot.

### Navigation performance in 2 patterns

In the online experiment, all 8 subjects could successfully complete the given task, which approaches the destination with obstacle avoidance via 2 control patterns. In general, the design of the interface is sufficient to permit the user to operate the system correctly. The task duration, command times, path length, collision times, and effort level in different patterns are shown in Table 3.

**Table 3.** Evaluation of online experiment for each subject.

Subject ID	Task duration (s)		Command times		Path length (m)		Collision times		Effort level (1–5)	
	SS	CVT	SS	CVT	SS	CVT	SS	CVT	SS	CVT
S1	204.6	236.9	8.7	3.7	8.5	8.5	0	0	3	2
S2	349.4	307.0	10.0	3.3	8.4	8.9	0	0	4	1
S3	309.4	250.6	11.3	3.7	9.8	9.1	0	0	5	1
S4	270.0	214.9	13.3	3.3	8.5	9.1	1	0	3	3
S5	483.1	276.3	13.3	3.7	9.5	10.7	0	0	4	2
S6	323.1	281.6	12.3	3.7	9.2	7.6	0	1	4	2
S7	331.4	242.1	10.7	3.3	8.2	9.3	1	0	5	3
S8	334.9	204.9	9.7	3.0	7.7	8.5	1	1	3	2
Mean	325.7	251.8	11.2	3.5	8.7	9.0	0.4	0.3	4.1	2.0
SD	78.8	34.7	1.7	0.3	0.7	0.9	0.5	0.4	0.8	0.7

For the mean task duration of all subjects, the CVT pattern reduces the task duration significantly from 325.7 ± 78.8 s to 251.8 ± 34.7 s (*P* < 0.05), as compared to SS, which saves 22.7% of the time. The task duration of CVT is obviously shorter than the SS for 7 subjects, and only S1 takes longer to perform the CVT than the SS, which may be related to his excellent decision during the SS task. He completed the SS task in only 204.6 s, which is the shortest task duration among all subjects. The results show that the CVT is obviously more time efficient for most subjects.

The command times showed a similar reduction, with a mean value from 11.2 ± 1.7 in SS to 3.5 ± 0.3 in CVT, which saves approximately 67.4% command times. All subjects are able to complete the avoidance of 2 obstacles with 3 to 4 commands by CVT, while it takes 8 to 12 commands to complete by SS. Subjects showed more individual differences in SS compares to CVT. Therefore, the CVT can decrease command output markedly.

The path lengths and collision times of the 2 experiments are barely much different. Five subjects travel a shorter path length in SS mode while 3 subjects travel a shorter path length in CVT. Three subjects collided with obstacles a total of 3 times under the SS pattern, and 2 subjects collided with obstacles a total of 2 times under the CVT task. No one bumps the baffle in the entire experiment. CVT does not help much in shortening path lengths and reducing collisions in this scenario.

All subjects agreed that the CVT pattern exerted less effort level. According to the subjective evaluation, the CVT pattern is an easier control pattern that required less workload than the SS pattern. The supervisor evaluation shows that the CVT pattern is easy to use. Overall, it is feasible for the user to assist the robot in avoiding undetected obstacles in both patterns. Compared to the SS pattern, the CVT pattern gives the user less operational burden and faster control.

### Navigation trajectory in 2 patterns

**Fig. 8.**
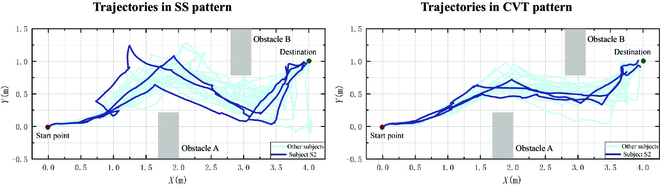
Navigation trajectories of 8 subjects in SS and CVT patterns.

Navigation trajectory is an essential index to evaluate whether the CVT is performing as expected. Figure 8 illustrates the navigation trajectories of 8 subjects in different control patterns. The gray squares represent 2 extremely low obstacles. The red and green dots refer to the robot's start point and destination. The coordinate of the start point is (0, 0) and the coordinate of the destination is (4.0, 1.0). An ideal length of the navigation path shown in Fig. 7A is 7.8 m. The purple trajectories indicate the driving path of subject S2 and the blue trajectories indicate the driving path of the other 7 subjects.

From an overall perspective, the trajectories of all the subjects performing in the SS pattern are more divergent, while the trajectories are more clustered in the CVT pattern. This implicates that subjects chose more similar paths in the CVT pattern. Subjects stopped an average of 3.5 times and 11.2 times for the navigation track in the CVT and SS pattern, separately. The trajectory in CVT is apparently smoother and has smaller angle changes than SS. Trajectories corresponding to the SS pattern are regarded as a broken line, due to more pauses during the robot motion. In contrast, trajectories corresponding to the CVT pattern appear more coherent, due to fewer pauses. SS generates more situations where the distance to baffles or obstacles is too close. All collisions occurred on obstacle B in 2 patterns, and most subjects have a closer distance to obstacle B than A. This may be related to the fact that obstacle B is close to the destination. The ratio of the average path length to the ideal trajectory length is 1.2 in the CVT pattern, and it achieves 1.1 in the SS pattern. The conjecture for this result is that subjects adopted a closer-to-obstacles approach to avoid obstacles in the SS pattern. In the CVT pattern, subjects tend to use a more secure approach and adopt more distant target positions to avoid obstacles.

## Discussion and Conclusion

Humans making macro decisions and robots executing fine movements are the major approach adopted by most shared control systems. It reduces the burden on users while increasing efficiency. The method of navigation goal selection is the key to providing free interaction. In this study, we adopt the CVT approach to generate navigation goals that cover all optional goals and varied according to the environmental changes. The CVT-based road selection method provides a more free and comprehensive choice. In Ref. [40], MI and P300 potential were combined to control a wheelchair asynchronously. It took 123.8 s to travel 4.7 m without obstacle avoidance, and the subjects issued 3 commands. In Ref. [26], real-time reconstruction of the scenario was performed in the virtual evoking paradigm. It projects navigation goals to the BCI paradigm. It took 571 s to travel 15.7 m with 2 radar-detectable obstacle avoidances. In this study, it took approximately 3.5 commands for the user assisting the robot to complete 2 undetectable obstacle avoidances, and it took 251.8 s to travel a path of 9 m. With the same average speed, our system enables the user to intervene to avoid undetectable obstacles. Our system has an advantage in case of decision errors by the robot. In the future, we will further optimize the algorithm to achieve speed-controlled navigation. The navigation algorithm would be optimized to cope with multiple environments where robots are prone to make poor decisions.

Asynchronous control is an essential aspect of practical applications. It allows the user to intervene in the system at self-pace. The judgment basis for state switching is the key to asynchronous control. Here, we combine EMG and EEG signals for asynchronous control. This combination integrates the high accuracy of ERP and the rapid response of EMG. The EMG signals generated by clenching teeth are distinguished well and the ERP of EEG is detected efficiently. This model ensures that the user controls the robot anytime, and it still functions properly when users exit control. In Ref. [40], the authors issued the start command and stop command with an average time of 4.06 s and 3.0 s by MI and P300 potential. In Ref. [41], the stop command was generated by eye blinking in an average of 3.0 s. In our study, the EMG signals were used to generate the intervention command with an average time of 2.7 s. The EMG signals are fast enough as a state switch.

This study proposed a CVT-A-based BCI system to achieve brain-controlled robot navigation with the obstacle avoidance task. The hybrid asynchronous system incorporates ERP in the EEG signals and EMG signals created by teeth clenching for self-paced control. Furthermore, a novel road segmentation method based on CVT was used to generate optional navigation goals for arbitrary target selection. The robot combines the commands obtained from the human with the autonomous navigation function to reach the human-selected goals. A comparison experiment was conducted that demonstrated that the proposed system can optimize navigation paths and reduce operational burden.

## Data Availability

The data that support the findings of this study are available upon reasonable request from the authors.
